# Mothers’ Satisfaction Rate from Hospital Cares in Hematology- Oncology Ward 

**Published:** 2015-07-20

**Authors:** H Boroumand, M Moshki, A Khajavi, H Hashemizadeh

**Affiliations:** 1 Dr Sheikh hospital, Mashhad University of Medical Sciences, Mashhad, Iran.; 2 Associate Professor, Department of public Health, School of Health; Social Development & Health Promotion Research Center, Gonabad University of Medical Sciences, Gonabad, Iran.; 3 Department of Community Medicine, School of Medicine, Gonabad University of Medical Sciences, Gonabad, Iran.; 4 Social Development and Health Promotion Research Center, Gonabad University of Medical Sciences, Gonabad, Iran.; 5 Department of nursing, Quchan Branch, Islamic Azad University, Quchan, Iran.

**Keywords:** Children, Hematology-Oncology Ward, Hospital Care, Mothers Satisfaction

## Abstract

**Background:**

Satisfaction evaluation is a good way to assess hospital conditions. In Health Care System, parentscan be also as children's main supporters, thus they may act as patient's viewpoints' representatives.This study aimed to evaluate mother’s satisfaction of hospital care in hematology – oncology ward in Dr Sheikh hospital.

**Materials and Methods:**

A Cross-sectional descriptive analytic study was conducted using Pediatric Family Satisfaction (PFS) questionnaire and interviewing with 164 mothers duringMarchto February2013. The obtained data were analyzed using SPSS -16 software and descriptive statistics.

**Results:**

The mean age of mothers and children was31.2±5.8, and 7.95 4/66 years.The children were 64 % male and 36 % femael. A large number of mothers (%56 (describedtheir satisfaction about medical care as moderate,(%70.7) reported their satisfaction about nursing care at very high level and(36.5 %) reported satisfaction about welfare services at high level(59%)and describe overall satisfaction at very high level . The totals mean of mothers’ satisfaction ratewas 121.8 ± 10.8. The mean of medical care, nursing care, welfare services was 2.9±34.1,4.6±50 and4.8± 32.9 respectively.

**Conclusion:**

Overall satisfaction with medical, nursing and welfare staff was acceptable. For more satisfaction, it is widely recommended to improve veinipuncture by nurses, Physicians should inform parents about the tests results, and finally disturbance in ward with noise should be controled.

## Introduction

New movement in health industries have been initiated towards continuous quality improvement and this phenomenhas gained momentum since 1990 .According to Donabedian's declaration for incorporating patient perception into quality assessment, healthcare managers incorporate patient centered care as a major component in the healthcare mission(1). The healthcare managers who endeavor to achieve excellence take patient perception into account when designing the strategies forquality improvement of care. Recently, the healthcare regulators shifted towards a market -driven approach of turning patient satisfaction surveys into a quality improvement tool for overall organizational performance (2). In 1996, evaluation of patient satisfaction was mandatory for all French hospitals (3). Since 2005, measuring satisfaction has been considered as an obligatory element of quality management reports in Germany(4).Since 2002, the Department of Health (DOH) has launched a national survey program in which all NHS trusts in England have to seek patient satisfaction on an annual basis and report the obtained results to their regulators(5). Therefore, measurement of patient satisfaction is a legitimate indicator for improving the services and strategic goals for all healthcare organizations (6).The most important outcome of pediatric care is the improvement of the child’s health or reduction of symptoms. However, parents’ satisfaction is associated with such central outcomes, including adherence to the therapeutic regimen and understanding of medical information, parents’ satisfaction with care can be considered a good proxy variable for some important aspects of quality care (7). Unfortunately, until now, parental experience with pediatric inpatient care has not been carefully explored. (8).In general, satisfaction is consideredas as a key measure in care quality; however, different attitudes toward the prominence of various issues are not commonly included in the studies. (9).Satisfaction evaluation is a good way to assess hospital conditions. In Health Care System, parents considered as children's main supporters, thus they may act as the patient's viewpoints' representatives.Thisstudy aimed to evaluate mother’s satisfaction of hospital care in hematology – oncology ward in Dr Sheikh hospital.

## Materials and Methods

A Cross-sectional descriptive analytic study was conducted using Pediatric Family Satisfaction (PFS) questionnaire and interviewing with 164 mothers during 2013. The interview was done after discharging from the hospital.

Questionnaire: The questions reflected 3 dimensions: medical care (9 items), nursing care (11 items), and welfare services (8 items). For each item, the parents were asked to assess what they found most important on a 5-point Likert scale, from excellent to not applicable. The questionnaire also included items on mother characteristics: age and education. The pilot studywasdone and had a pretest of questionnaire in 15 parents.For reliability we use internal consistency. The Cronbach α score for 3 dimensions was 0.9. The distribution ofthe responses were normal.

To describe Motherssatisfaction Rate from hospital cares, the mean score of each item was calculated using scores from 1 to 5 on the rating scale. Statistical analysis was carried out using SPSS 16 statistical software (SPSS Inc, Chicago, Ill). Statistical significance was set at P<.05.We also used Anova and t- test. 

## Results

The mean age of mothers and children were 31.2±5.8 and 7.95 4.66 years, respectively. The children were 64 % male and 36 % female. A large number of mothers(%56 (described their satisfaction as follow:about medical care as moderate,(%70.7) about nursing care at very high level, and(36.5 %) about welfare services at high level(59%)and very high level about all aspect. The total mean of Mothers’ Satisfaction Rate was 121.8 ± 10.8 .The mean of medical care, nursing care, and welfare services were2.9 ± 34.1 406±50and4.8± 2.9, respectively.Regarding medical care The main points, evaluated weakly, in order of importance included as follows: to inform parents about the tests results (29.9 %), regarding nursing care :doing veinipuncture (4.9%) and in regard to welfare services: disturbance in ward with noise(1.8%). There is a negative relationship between mother's education and mother's satisfaction (ANOVA, p=0.007).

**TableI:** Frequency of number of children in family

**number of children**	**%**	**number**	**%**
**one**	31.1	51	31.1
**two**	43.3	71	43.3
**tree or more**	25.6	42	25.6
**Total**	100	164	100

**TableII:** Frequency of ratingbirth

**Ratingof birth**	**number**	**%**
**one**	77	47.0
**two**	65	39.6
**tree or more**	22	13.4
**Total**	164	100

**TableIII:** Frequency of Residence

**Residence**	**number**	**%**
**city**	109	66.5
**Rural**	55	33.5
**Tatal**	164	100

**TableIV:** Frequency type of malignancy

**Type of malignancy**	**%**	**number**	**%**
**Leukemia**	**53**	**86**	**53**
**Lymphoma**	**15**	**25**	**15**
**Nephroblastoma**	**10**	**17**	**10**
**Neuroblastoma**	**8**	**13**	**8**
**Others**	**14**	**23**	**14**
**Total**	**100**	**164**	**100**

**Table V:** Frequency of Mothers’ satisfaction in Relation to Medical Care

**satisfaction** **Question**	**Excellent**	**Good**	**Fair**	**Poor**		**not applicable**	**Total**
**1-** ** were caring and concerned**	128 (78 )	29 ( 17.7)	7 ( 4.3)		-	-	164 (100 )
**2- were familiar with the medical history**	116 (70.7)	38 ( 23.2 )	9 (5.5)		1 ( 0.6 )	-	164 (100 )
**3-were available when needed or called**	101 (61.6)	53 ( 32.3)	7 ( 4.3)		3 (1.8 )	-	164 (100 )
**4- ** **responded promptly to changes in the patient's condition.**	105 ( 64)	47 (2807 )	10 ( 6.1)		2 ( 1.2)	-	164 (100 )
**5-** ** had clear, honest communication with us**	120 ( 73.1)	34 ( 20.7 )	9 ( 5.4 )		1 ( 0.6 )	-	164 (100)
**6-** ** gave us information about treatments and tests before they were done**	75 ( 45.7)	35 (21.3 )	11 (6.7 )		18 (11)	25 (.15.2 )	164 (100 )
**7- ** **kept us informed of test results an of changes In the patient's condition**	49 (29.9)	16 (9.8 )	21 (12.8 )		29 (17.7 )	49 (29.9 )	164 (100 )
**8- ** **gave us complete explanations**	103 (62.8)	38 (23.2)	20 (12.2)		1 (0.6 )	2 (1.2)	164 (100 )
**9- ** **answered our questions clearly**	108 (65.9 )	45 (27.4)	9 (5.5)		2 (1.2)	-	164 (100 )

**Table VI:** Frequency of Mothers’ satisfaction in Relation to Nursing Care

**satisfaction** **Question**	**Excellent**	**Good**	**Fair**	**Poor**	**not applicable**	**Total**
**1-** ** were caring and concerned**	131 ( 79.9)	29 (17.7 )	4 (2.4 )	-	-	164 (100 )
**2-** ** were gentle with the patient**	129 (78.7 )	29 ( 17.7)	5 (3 )	1 ( 0.6)	-	164 (100 )
**3- ** **Checked the patient's condition closely**	113 ( 68.9)	46 ( 28)	3 ( 1.8)	2 ( 1.2)	-	164 (100 )
**4- ** ** notified the doctor when necessary**	138 (84.1 )	24 (14.6 )	2 ( 1.2)	-	-	164 (100 )
**5- ** **Gave treatment and medication on time**	119 (72.6 )	40 ( 24.4)	3 ( 1.8)	2 ( 1.2)	-	164 (100 )
**6-** ** kept us informed**	83 ( 50.6)	65 ( 39.6)	11 ( 6.7)	5 (3 )	-	164 (100 )
**7-** ** Answered our questions clearly**	93 (56.7 )	59 ( 36)	10 ( 6.1)	2 (1.2 )	-	164 (100 )
**8-** ** explained the patient's condition and care in terms we understood**	80 (48.8 )	55 (33.5 )	22 ( 13.4)	7 (4.3 )	-	164 (100 )
**9- themethod ofvenipuncture is suitable **	66 ( 40.2)	71 (43.3 )	19 ( 11.6)	8 (4.9 )	-	164 (100 )
**10- reducepainwhen required**	94 (57.3 )	56 (34.1 )	10 (6.1 )	4 (2.4 )	-	164 (100 )
**11- reducefeverwhen required**	105 (64 )	46 (28 )	11 (6.7 )	2 (1.2 )	-	164 (100 )

**Table VII:** Frequency of Mothers’ satisfaction in Relation to welfare services

**satisfaction** **Question**	**Excellent**	**Good**	**Fair**	**Poor**	**not applicable**	**Total**
**1. The admission procedure went smoothly. **	93 (56.7 (	51 )31.1 (	16 )9.8 (	4 (2.4 (	-	164 (100 )
**2- The unit and patient rooms were clean**	98 )59.8 (	40 )24.4(	24 )14.6 (	2 )1.2 (	-	164 (100 )
**3- The decor and furnistiings were suitable. **	79 )48.2 (	46 )28 (	27 )16.5 (	11 )6.7 (	1 (0.6 )	164 (100 )
**4- The patient was not disturbed by noise. **	51 )31.1 (	51 )31.1 (	39 )23.8 (	20 )12.2 (	3 (1.8 )	164 (100 )
**5- provided age-appropriate toys, games, play- rooms, and activities**	82 )50 (	42 )25.6 (	25 [)15.2 (	13 )7.9 (	2 (1.2 )	164 (100 )
**6- The hospital staff worked together as a team.**	100 (61 (	45 )27.4 (	17 )10.4 (	2 )1.2 (	-	164 (100 )
**7-Availabilityphonesneeded**	50 )30.5 (	59 )36 (	30 )18.3 (	24 )14.6 (	1 (0.6 )	164 (100 )
**8- The qualityof food**	58 )35.4 (	44 (26.8 (	19 )11.6 (	43 )26.2 (	-	164 (100 )

**Table VIII:** Frequency of Mothers’ satisfaction in Relation to mean of medical care,nursing care and welfare services

**Satisfaction** **Type of care**	**Mean**	**SD**	**Min**	**Max**
**medical care**	34.1	2.9	26	40
**nursing care**	50	4.6	35	55
**welfare** ** services**	32.9	4.8	20	40
**Total**	121.8	10.8	95	140

**Table IX:** Frequency of Mothers’ satisfactionrate

**Satisfaction** **Type of care**	**Excellent**	**Good**	**Fair**	**Poor**	**not applicable**	**Total**
	n (%)	n (%)	n (%)	n (%)	n (%)	n (%)
**medical care**	-	62 (38)	92 (56)	10 ( 6 )	-	164 ( 100)
**nursing care**	116 (70.7)	37 (22.5)	11 (6.7)	-	-	164 ( 100)
**welfare** ** services**	59 (36)	60 (36.5)	25 (15.3)	18 (11)	2 (1.2)	164 ( 100)
**Total**	97 (59)	52 (32)	15 (9)	-	-	164 ( 100)

## Discussion

Hosseinian and et al in their research about Mothers satisfaction of hospital care in the pediatric ward of Kashan reported mothers were dissatisfied with doctors for not being available on time and their failure to notify the results of their chidern’stests. They were also dissatisfied with nurses for the lack of education about their chidern’streatment and also the lack of post-discharge care. The mothers were also dissatisfied with welfare services (e.g. providing an appropriate play room for children (71.4%) Average satisfaction scores for the medical, nursing,and welfare staff were 22.25±6.19, 29.05±6.88,:and 26.68±6.93,respectively. A significant relationship was observed between child's disease and mothers satisfaction (P<0.0001) (9).

Pourmovahed and etal in their research titled as Mothers satisfaction Rate of Hospital Cares in the Pediatric Ward at Sadoqi Hospital of Yazd reported the main points, regarding medical care, evaluated weakly, in order of importance included as follows: to inform parents about the tests results and the medical managements (9%), regarding nursing care: to aware parents about medical care and post-charge care (10.5%) and then in regard to welfare services: availability of playing conditions for children in the ward (56.5%). Overall mean score for welfare, nursing,and medical care were 32.01±4.58, 47.32±7.43and 38.36±6.13respectively (10).

Failure to notify the results of their chidern’stests by doctors was one of the finding reported in this study which is in line with other studies such as Hosseinian and Pourmovahed. 

The results of tests are very vital in oncology ward and doctors should report the results of tests to parents.

The main points, regarding nursing care and welfare services were doing veinipuncture (4.9%) and disturbance in ward with noise(1.8%). veinipuncturein oncology ward is a important role of nurses and it should be done by expert nurses because most of the children havedamaged vein caused by chemotherapy.

Noise disrupts the peace of mothers and their children and since psychological state of them is not stable, the noise can make bad Situation for them. ; therefore, noise control is essential in the ward. In this study, overall satisfaction in children with mother's education with bachelor's degree was lower (ANOVA, p=0.002).The obtained findings revealed that

There is a negative relationship between mother's education and mother's satisfaction.Pourmovahed and etal in their researchshowed the same) (10).

## Conclusion

Overall satisfaction reported excellent in oncologic ward of this hospital.To satisfy mothers and improve their satisfactionit is necessaryto inform parents about the tests results, veinipuncture should be done by expert nurses and thenoiseshould be control in ward. There is a growing interest in mother's satisfaction as an outcome of care and as an indicator of the quality of care. Interest in mother's satisfaction with pediatric care is not new, but research in this field is limited.
